# Effects of prenatal food and micronutrient supplementation on child growth from birth to 54 months of age: a randomized trial in Bangladesh

**DOI:** 10.1186/1475-2891-10-134

**Published:** 2011-12-08

**Authors:** Ashraful Islam Khan, Iqbal Kabir, Eva-Charlotte Ekström, Kajsa Åsling-Monemi, Dewan Shamsul Alam, Edward A Frongillo, Md Yunus, Shams Arifeen, Lars-Åke Persson

**Affiliations:** 1 International Maternal and Child Health, Department of Women's and Children's Health, Uppsala University, Sweden; 2 International Centre for Diarrhoeal Diseases Research, Bangladesh (ICDDR,B), Bangladesh; 3 Department of Health Promotion, Education and Behavior, Arnold School of Public Health, University of South Carolina, Columbia, USA

**Keywords:** Child growth, food supplementation, multiple micronutrients, pregnancy, programming, stunting

## Abstract

**Background:**

There is a lack of information on the optimal timing of food supplementation to malnourished pregnant women and possible combined effects of food and multiple micronutrient supplementations (MMS) on their offspring's growth. We evaluated the effects of prenatal food and micronutrient interventions on postnatal child growth. The hypothesis was that prenatal MMS and early invitation to food supplementation would increase physical growth in the offspring during 0-54 months and a combination of these interventions would further improve these outcomes.

**Methods:**

In the large, randomized MINIMat trial (Maternal and Infant Nutrition Interventions in Matlab), Bangladesh, 4436 pregnant women were enrolled between November 2001 and October 2003 and their children were followed until March 2009. Participants were randomized into six groups comprising 30 mg Fe and 400 μg folic acid (Fe30F), 60 mg Fe and 400 μg folic acid (Fe60F) or MMS combined with either an early (immediately after identification of pregnancy) or a later usual (at the time of their choosing, i.e., usual care in this community) program invitation to food supplementation. The anthropometry of 3267 children was followed from birth to 54 months, and 2735 children were available for analysis at 54 months.

**Results:**

There were no differences in characteristics of mothers and households among the different intervention groups. The average birth weight was 2694 g and birth length was 47.7 cm, with no difference among intervention groups. Early invitation to food supplementation (in comparison with usual invitation) reduced the proportion of stunting from early infancy up to 54 months for boys (p = 0.01), but not for girls (p = 0.31). MMS resulted in more stunting than standard Fe60F (p = 0.02). There was no interaction between the food and micronutrient supplementation on the growth outcome.

**Conclusions:**

Early food supplementation in pregnancy reduced the occurrence of stunting during 0-54 months in boys, but not in girls, and prenatal MMS increased the proportion of stunting in boys. These effects on postnatal growth suggest programming effects in early fetal life.

**Trial registration number:**

ISRCTN: ISRCTN16581394

## Background

Epidemiological and experimental studies indicate fetal under-nutrition affects large numbers of infants in developing countries, with adverse consequences for their immediate survival and lifelong health [1,2]. Studies in India have shown that maternal nutrition is an important determinant of fetal growth, size at birth, and post natal growth [3], and highlight the need for improving maternal diet through micronutrient rich foods [4]. The diets of women in low- and middle-income countries are frequently deficient in energy, protein, and different micronutrients [5]. The usual diet in rural Bangladesh is monotonous and low in energy calories [6]. Rice is the staple food, and is usually eaten with green-leafy vegetables and sometimes a small amount of fish. Consumption of meat and other animal products is uncommon. Maternal energy intake at 5 to 7 months of pregnancy is reportedly only 1464 kcal/day [7].

A Cochrane review [8] concludes the provision of balanced energy and protein supplements to undernourished pregnant women results in a small increase in birth weight (mean difference +38 g, 95% CI 0 to +75 g) and fewer stillbirths. The low amount of food intake of poor dietary quality combined with increased prenatal nutrient requirements for placental and fetal growth can result in multiple micronutrient deficiencies that may adversely affect pregnancy outcomes [8,9]. Prenatal multiple micronutrient supplements (MMS) were therefore developed and recommended for trial purposes [10]. A meta-analysis [11] has shown that multiple micronutrients when supplied during pregnancy in low- and middle-income countries resulted in a small increase in birth weight (mean increase +22 g, 95% CI 8.3 to 36 g) and a reduction in the occurrence of low birth weight. In a randomized trial in Madura, East Java, a prenatal high-energy supplementation resulted in postnatal effects; children were heavier up to the age of 24 months and increased height throughout the first 5 years more than children of mothers receiving a low energy supplement did [12]. Two recent studies have suggested that prenatal multiple micronutrient supplementation can result in a modest but increased growth up to two years of age [13,14]

There is a lack of information, however, about the effect of the timing of food supplementation during pregnancy on birth size and subsequent child growth. Animal experiments indicate the timing of under-nutrition in pregnancy produces different responses to size at birth, placental size, and development of endocrine responses [15]. Although specific human studies are scarce, a healthy diet starting from early pregnancy, or before, is important for the prevention of several adverse pregnancy outcomes [16,17]. Few studies address the possible effect of combining food supplementation with multiple micronutrients or the effect of fortifying food with multiple micronutrients. A randomized controlled trial in rural Burkina Faso [18] suggests prenatal multiple micronutrient-fortified food supplements results in longer birth length than just a multiple micronutrient supplement. The effect of the timing of food supplementation combined with different micronutrient alternatives on postnatal growth has not been well investigated.

The Maternal and Infant Nutrition Interventions in Matlab (MINIMat, trial registration ISRCTN16581394) is a randomized trial conducted in a rural community in Bangladesh. This trial evaluates the effect of early or later invitation to prenatal food supplementation, combined with one of three different micronutrient supplements, on anthropometry and gestational age at birth and under-five mortality (primary outcomes). The effect on child growth from birth to 54 months is reported in this paper.

The hypothesis was that prenatal multiple micronutrient supplementations and early invitation to food supplementation would increase physical growth in the offspring during 0-54 months, and a combination of these interventions would further improve these outcomes. A factorial design was used to determine the effect of a combination of prenatal micronutrient supplementation (daily doses of 60 mg iron and folate, 30 mg iron and folate or multiple micronutrients including 30 mg iron and folate) and early (around week 9) or later usual standard invitation (usually in the second trimester) to food supplementation on physical growth (weight and length/height) in the offspring during 0-54 months in a poor rural population in Bangladesh.

## Methods

### Participants

The study, Maternal and Infant Nutrition Interventions in Matlab (MINIMat), was conducted in Matlab, a rural sub-district 57 km southeast of the capital, Dhaka, Bangladesh. In this area, most people depend on agriculture, fishing, or day labor. Breast-feeding is almost universal during infancy. According to the national policy, children from 6-59 mo should be supplemented with vitamin A every 6 months and mothers should receive a vitamin A dose within 2 months after delivary. In Matlab, ICDDR,B maintains a Health and Demographic Surveillance System that has recorded health and demographic information on a monthly basis since 1966. In half of the area ICDDR,B is service provider for maternal and child health. All pregnant women in the ICDDR,B service area were eligible for enrolment. If a woman reported to the Community Health Research Worker (CHRW) that she was pregnant, she was offered a pregnancy test. She was enrolled in the MINIMat study if the following eligibility criteria had been met: fetus was viable, gestational age was < 14 weeks confirmed by ultrasound examination, no severe illness, and consent to participate. In total, 4436 pregnant women were enrolled in the MINIMat study between November 2001 and October 2003, and their children were followed-up until March 2009.

### Ethics

Written informed consent was obtained from all participating mothers, and the study was approved by the Research and Ethical Review Committees of ICDDR,B.

### Interventions

Details of the food and micronutrients supplementation are described elsewhere [19]. In brief, a factorial design was employed. Enrolled pregnant woman were randomly assigned to two food supplement groups: (early invitation: immediately after identification of pregnancy or later usual invitation: at the time of their choosing, i.e., usual care in this community in the second trimester) and three micronutrient groups: capsules containing 30 mg Fe fumarate + 400 μg folate (Fe30F); standard program 60 mg Fe fumarate + 400 μg folate (Fe60F); or, MMS (including 30 mg iron and folate). The MMS group received 15 different vitamins and minerals. Each MMS capsule contained 150 μg I (potassium iodide); 15 mg Zn (sulfate); 65 μg Se (sodium selenite); 2 mg Cu (sulfate); 800 μg retinyl acetate (RE) vitamin A; 1.4 mg thiamine mononitrate; 1.4 mg vitamin riboflavin; 18 mg vitamin B-3 (niacin); 1.9 mg vitamin B-6 (pyridoxine hydrochloride); 2.6 μg vitamin B-12 (cyanocobalmin); 70 mg vitamin C; 200 IU vitamin D (vitamin D3); 10 μg vitamin E (α- tocopherol acetate); 30 mg Fe (fumarate); and, 400 μg folate. This resulted in six food and micronutrient groups. Food supplementation was supplied by an ongoing government-supported national program, which provides energy-protein supplement to all pregnant women, and was available through community nutrition centres (CNC) 6 d/week. In this trial, the food supplement was offered to all pregnant women irrespective of their nutritional status, assessed by body mass index, even though in the national program, only pregnant women with BMI < 18.5 kg/m^2 ^were supposed to receive the food supplements. The food supplement contained 80 g roasted rice powder, 40 g roasted pulse powder, 20 g molasses and 12 mL (6 g) soybean oil, and was provided in plastic packets that were to be mixed with water. The supplement provided 608 kcal (2.85 mJ) and 18 g vegetable protein and was continued up to end of pregnancy. The three types of micronutrient supplements were distributed in special pill bottles that looked identical and contained 35 capsules and were distributed in the homes during monthly home visits by the interviewers. The micronutrient supplements were offered to the enrolled women during a clinic visit at 14 weeks of gestation.

### Adherence to supplement intake

At every monthly home visits, the interviewers asked a series of questions to assess compliance with food supplementation in the previous 30 days. The micronutrient bottles in the study were equipped with a microprocessor inside the cap (eDEM) that recorded the date and time of every opening of the cap. This information was downloaded into a computer when the bottles were collected from women. The capsule-counting eDEM is regarded as the best available method for measuring adherence [20].

### Randomization

The enrolled pregnant women were individually randomized into one of six groups of food and micronutrient intervention by a computer-generated register of study identity numbers and random assignment of food and micronutrient groups. The micronutrient supplementation was double-blinded but the food supplementation was allocated randomly and not masked.

### Sample size

Sample size calculations were primarily done in relation to a birth weight outcome that had a standard deviation of 400 g, based on previous data from Bangladesh, and the minimum important difference was determined to be 70 g. The estimated sample size with a power of 0.90 (Type II error of 0.10) and 95% confidence level (type I error of 0.05) was 686 in each group, i.e., 4116 in the six food and micronutrient supplementation groups. Adjusting for 5% refusal, 11% loss during pregnancy, and 9% loss during infancy due to death and out-migration, the total sample size required was 5300. This required enrolment over two years at the known rates of pregnancy occurrence in this study population.

Because the MINIMat study was designed to evaluate the impact of nutritional interventions on birth weight, the sample size calculations were made on the basis of finding a difference in birth weight. This paper reports a secondary outcome (child growth). We calculated the differences in child growth that we could detect with the adequate sample size (n = 2735) in any supplementation group in our analyses. For this sample size, 80% power and 95% probability we were able to detect a difference of 0.2 SD score (or difference of 0.12 SD score for 2 food groups) between 6 food and micronutrient supplementation groups.

### Anthropometric outcomes

Anthropometry at birth was part of the primary outcome of this trial, and in this paper subsequent child growth is reported. Weight and length/height were measured every month up to 1 year, then every 3 months up to 24 months, and again at 54 months of age. Birth anthropometry was performed usually within 72 hours after birth. All birth weights were measured by SECA electronic or beam scales (SECA Gmbh & Co, Hamburg, Germany) to a precision of 10 grams. Maternal weight and height were measured on enrollment at around the eighth week of gestation. Maternal weight was measured by electronic scales (Uniscale, UNICEF, Copenhagen), with a precision of 100 grams, and height was measured to the nearest 0.1 cm with a stadiometer. The recumbent length of the newborn was measured by locally manufactured, collapsible length boards, with a precision of 1 mm. Weighing equipment was calibrated daily with standard weights, and refresher training was conducted periodically on data collection methods and anthropometric measurements. At the 54 month follow-up, body weight was recorded to the nearest 0.1 kg with a digital scale (TANITA HD - 318, Tanita Corporation, Japan) and with the child in light clothing and bare feet. The scale was checked on each study day with a standard 20 kg weight. After removing shoes, height was measured to the nearest 0.1 cm with a daily-calibrated freestanding stadiometer Leicester Height Measure (Seca 214, UK). Weight and length/height measurements were converted to weight-for-age, length/height-for-age, and weight-for-length/height Z-scores (SD scores), according to the WHO Multicentre Growth Reference Study child growth standards [21]. For this conversion, Anthro 2007 was used (WHO Anthro for personal computers, version 2, 2007: Software for assessing growth and development of the world's children. Geneva: WHO, 2007 http://www.who.int/childgrowth/software/en/). Underweight was defined as <-2 Z-score for weight-for-age, stunting was defined as <-2 Z-score for length/height-for-age, and wasting was defined as <-2 Z-score for weight-for-length/height.

### Socioeconomic measurements

All socioeconomic information was collected during a home visit at the time of enrollment. This information included household structure and family characteristics such as age of mother, parity, parental education, and employment. Data collectors had at least 10 years of education. Data were collected with structured questionnaires, which included both pre-coded and open ended questions and reviewed once every 2 week by the responsible investigators. Refresher training of the interviewers on methods to collect data and anthropometric measurements were repeated every 3 month.

### Analysis

An initial dataset was selected with those having the birth and 54 month measurements. Measurements at 1 and 2 months were excluded from analysis because data were missing for one-third of the cases. The data was interpolated linearly if one, or maximum two, consecutive measurements were missing. For example, if the 4-month weight was missing between the 3- and 5-months measurements, it was imputed from the average of the 3- and 5-month weights. For two consecutive missing values, the values were imputed from the weighted average of the previous and subsequent measurement values. Data imputation was done for missing values for continuous covariates such as weight and length/height and the SD scores were calculated. Finally, the data set included data for 16 anthropometric assessments: birth, and 3, 4, 5, 6, 7, 8, 9, 10, 11, 12, 15, 18, 21, 24 and 54 months.

The baseline and follow-up characteristics, including socioeconomic status indicators, maternal literacy and education, occupation, maternal nutritional status, age, and parity were compared across intervention groups. All singleton newborns were included in the intention-to-treat analysis. Means and standard deviations (SD) were calculated for continuous variables, and proportions were calculated for categorical variables. Histograms were used to confirm that continuous dependent variables were Gaussian, and the standard deviations of intervention groups were compared to ensure that they were similar. Differences between categorical variables were compared by chi-square tests. T-tests and analysis of variance with post hoc Bonferroni corrections were used to compare group differences. General linear modeling of repeated-measurements ANOVA was used to compare the different supplementation groups, mean height-for-age Z-scores and occurrence of stunting throughout the 54 months follow-up. Stunting prevalence ranged from about 17% (at birth) to 52% (month 21), and the linear response function could be used instead of the logistic response function. Occasion was the within-subject factor and the food and/or micronutrient supplementation group was the between-subject factor. Most statistical analyses were performed with SPSS (version 18.0; SPSS Inc., Chicago, IL, USA), while GraphPad (Prism 5 for Windows, version 5.04) software was used for the graphs. We considered results where p < 0.05 statistically significant and p = 0.05 as marginally significant.

## Results

**Figure 1:**
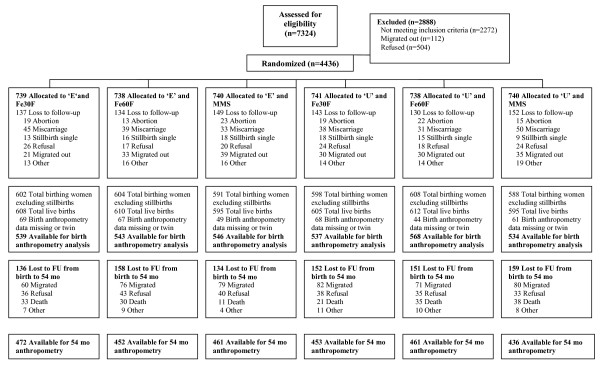
**Flow diagram of women and infants participating in the MINIMat trial**. E = Early invitation food supplementation, U = Usual invitation food supplementation, FU = Follow-up; Fe30F = 30 mg iron and 400 μg of folic acid; Fe60F = 60 mg iron and 400 μg of folic acid; MMS = multiple micronutrients, 15 micronutrients including 30 mg iron and 400 μg of folic acid;

There were 3267 singleton infants with birth anthropometry born by the 4436 women enrolled into the MINIMat trial. Losses before measurement of birth anthropometry included early fetal loss (n = 347), stillbirth (n = 89), out-migration (n = 188), and refusal to participate (n = 129). Birth anthropometry was missing for 358 children and 72 were excluded from analysis of birth anthropometry, as they were twins (Figure 1). Losses from follow-up after birth anthropometry up to the 54-months included out-migration (n = 448), refusal to participate (n = 225), infant or child mortality before 54 months (n = 168), and other reasons (n = 49). Losses to follow-up before birth anthropometry did not differ among the intervention groups (p = 0.676). The number of infant and child deaths between birth and 54 months differed, with a lower number of deaths in the early invitation-MMS group, which is reported elsewhere (Persson LÅ *et al. *manuscript submitted). Losses to follow-up after birth anthropometry, other than mortality, did not differ across the six intervention groups (migration p = 0.16 and refusal p = 0.55).

The anthropometric follow-up included a maximum of 18 occasions for weight and length/height measurements between birth and 54 months of age. The mean number of measurements was 14.3, with no difference across the intervention groups (p = 0.625). After imputation, an average of 15.4 (± 3.78) anthropometric measurements were available for analysis, and the number of measurements per child did not differ across the intervention groups (p = 0.60). For the longitudinal analysis, anthropometric information was available for 1634 children. The analyzed and non-analyzed groups were compared, and there was no evidence of selective dropout (p = 0.19) among the six intervention groups (data not shown). By design, early invitation to food supplementation consumed more packages of supplement than usual invitation to food supplementation (mean difference 30 packages from enrolment to wk 30 examination). On average the participants had taken 77 micronutrient capsules from wk 14 to 30. MMS groups had taken a few less capsules than the other groups (p = 0.025) and the early invitation food group also had a few less micronutrient capsules on the average (p = 0.015) (data not shown).

There were no significant differences in the characteristics of mothers and households across the different intervention groups (Table 1). The average/mean birth weight was 2693.9 g (± 410.5) and average birth length was 47.7 cm (± 2.2) with no significant difference across intervention groups (p = 0.345 for birth weight and p = 0.262 for birth length). Eight percent of the children were pre-term (born < 37 weeks of gestation) and 30% of the children were born with low birth weight (< 2500 g). These proportions did not differ across the intervention groups (p = 0.201 for pre-term and p = 0.143 for low birth weight).

**Table 1:** Baseline characteristics of mothers and households (n = 1634) with different supplementation groups in the MINIMat trial study

*Characteristics*	Early invitation food supplementation N (%)				Usual invitation food supplementation n (%)			
				
	Fe30F	Fe60F	MMS	All	Fe30F	Fe60F	MMS	All
**Age**								
< 20 years	38/258 (14.7)	43/291 (14.8)	36/290 (12.4)	117/839(13.9)	36/260(13.8)	34/275(12.4)	47/260(18.1)	117/795(14.7)
20-29 years	154/258 (59.7)	162/291 (55.7)	164/290 (56.6)	480/839(57.2)	146/260(56.2)	163/275(59.3)	147/260(56.5)	456/795(57.4)
≥ 30 years	66/258 (25.6)	86/291 (29.6)	90/290 (31.0)	242/839(28.8)	78/260(30.0)	78/275(28.4)	66/260(25.4)	222/795(27.9)
**BMI at 8 week (**kg/m^2^)								
< 18.5	68/256 (26.6)	81/290 (27.9)	80/289 (27.7)	229/835(27.4)	78/260(30.0)	76/274(27.7)	66/260(25.4)	220/794(27.7)
≥ 18.5	188/256 (73.4)	209/290 (72.1)	209/289 (72.3)	606/835(72.6)	182/260(70.0)	198/274(72.3)	194/260(74.6)	574/794(72.3)
**Education**								
No Education	78/258 (30.2)	85/291 (29.2)	104/290 (35.9)	267/839(31.8)	79/260(30.4)	97/275(35.3)	75/260(28.8)	251/795(31.6)
Education (read or write)	180/258 (69.8)	206/291 (70.8)	186/290 (64.1)	572/839(68.2)	181/260(69.6)	178/275(64.7)	185/260(71.2)	544/795(68.4)
**Parity**								
1	73/182 (40.1)	71/204 (34.8)	89/219 (40.6)	233/605(38.5)	76/190(40.0)	73/208(35.1)	77/178(43.3)	226/576(39.2)
2	61/182 (33.5)	77/204 (37.7)	61/219 (27.9)	199/605(32.9)	59/190(31.1)	79/208(38.0)	56/178(31.5)	194/576(33.7)
3 or more	48/182 (26.4)	56/204 (27.5)	69/219 (31.5)	173/605(28.6)	55/190(28.9)	56/208(26.9)	45/178(25.3)	156/576(27.1)
**Gestational age**								
< 37 weeks	14/258 (5.4)	17/291 (5.8)	20/290 (6.9)	51/839(6.1)	19/260(7.3)	16/275(5.8)	12/260(4.6)	47/795(5.9)
≥ 37 weeks	244/258 (94.6)	274/291 (94.2)	270/290 (93.1)	788/839(93.9)	241/260(92.7)	259/275(94.2)	248/260(95.4)	748/795(94.1)
**Household**								
**Socioeconomic status: quintiles**								
Q1+Q2	103/258 (39.9)	115/291 (39.5)	129/290 (44.5)	347/839(41.4)	103/260(39.6)	118/275(42.9)	106/260(40.8)	327/795(41.1)
Q3	65/258 (25.2)	57/291 (19.6)	57/290 (19.7)	179/839(21.3)	47/260(18.1)	57/275(20.7)	52/260(20.0)	156/795(19.6)
Q4+Q5	90/258 (34.9)	119/291 (40.9)	104/290 (35.9)	313/839(37.3)	110/260(42.3)	100/275(36.4)	102/260(39.2)	312/795(39.2)

Q1 lowest quintile (poor); Q5 highest quintile (rich)

Fe30F = 30 mg iron and 400 μg of folic acid; Fe60F = 60 mg iron and 400 μg of folic acid; MMS = multiple micronutrients, 15 micronutrients including 30 mg iron and 400 μg of folic acid;

Anthropometric indices at birth were low, gradually worsened up to 18 months, and then improved slightly at 54 month of age. For example, at birth, height-for-age Z-score (HAZ) was mean -0.91 and proportion of stunting was 17%; at 18 months, HAZ was mean -1.94 and stunting 49%; and, at 54 months mean was -1.49 and stunting 31%. There was no significant difference in mean weight-for-age, weight-for-height, or height-for-age (n = 1634) in the cross-sectional analyses across intervention groups (Table 2). Furthermore, the mean of the anthropometric measurements did not differ across intervention groups at 54 months (p = 0.240) when including all children (n = 2735) who were anthropometrically assessed at that age.

**Table 2:** Anthropometry among the different supplementation groups in the MINIMat trial study (n = 1634) in children from birth to 54 months

Nutritional status (Z score)	Early invitation food supplementation				Usual invitation food supplementation			
				
	Fe30F	Fe60F	MMS	All	Fe30F	Fe60F	MMS	All
**At birth**								
WHZ	-0.88 ± 1.11	-0.97 ± 1.16	-0.87 ± 1.01	-0.91 ± 1.09	-0.93 ± .99	-0.90 ± 1.02	-0.86 ± 1.01	-0.89 ± 1.01
HAZ	-0.89 ± 1.12	-0.83 ± 1.05	-1.01 ± 1.08	-0.91 ± 1.08	-0.94 ± 1.12	-0.93 ± 1.05	-1.01 ± 1.12	-0.96 ± 1.09
WAZ	-1.27 ± 0.96	-1.27 ± 0.94	-1.35 ± 0.95	-1.29 ± .95	-1.33 ± .99	-1.33 ± 0.89	-1.34 ± 0.92	-1.33 ± 0.93
**At 3 months**								
WHZ	-0.21 ± 1.01	-0.22 ± 1.03	-0.15 ± 1.16	-0.19 ± 1.07	-0.19 ± 1.28	-0.08 ± 1.14	-0.02 ± 1.12	-0.09 ± 1.18
HAZ	-1.31 ± 0.97	-1.28 ± 0.97	-1.37 ± 1.07	-1.32 ± 1.00	-1.32 ± 1.18	-1.37 ± 1.03	-1.46 ± 1.05	-1.38 ± 1.09
WAZ	-1.23 ± 0.99	-1.22 ± 0.96	-1.25 ± 0.97	-1.23 ± 0.97	-1.23 ± 1.12	-1.21 ± 1.04	-1.24 ± 1.03	-1.23 ± 1.06
**At 6 months**								
WHZ	-0.29 ± 1.09	-0.29 ± 1.02	-0.25 ± 1.05	-0.28 ± 1.05	-0.20 ± 1.18	-0.19 ± 1.05	-0.17 ± 1.12	-0.19 ± 1.12
HAZ	-1.23 ± 0.98	-1.34 ± 0.93	-1.37 ± 0.98	-1.32 ± 0.97	-1.36 ± 1.09	-1.36 ± 1.03	-1.51 ± 1.01	-1.41 ± 1.05
WAZ	-1.06 ± 1.04	-1.12 ± 0.98	-1.12 ± 1.01	-1.10 ± 1.01	-1.08 ± 1.17	-1.07 ± 1.06	-1.15 ± 1.07	-1.09 ± 1.10
**At 12 months**								
WHZ	-0.68 ± 1.03	-0.73 ± 0.95	-0.72 ± 1.03	-0.71 ± 1.00	-0.69 ± 1.19	-0.64 ± 0.95	-0.59 ± 1.09	-0.64 ± 1.08
HAZ	-1.59 ± 0.97	-1.65 ± 0.88	-1.72 ± 1.02	-1.65 ± 0.96	-1.67 ± 1.05	-1.73 ± 1.08	-1.78 ± 1.02	-1.73 ± 1.05
WAZ	-1.29 ± 1.05	-1.37 ± 0.95	-1.39 ± 1.09	-1.36 ± 1.03	-1.35 ± 1.17	-1.35 ± 1.05	-1.34 ± 1.09	-1.35 ± 1.11
**At 18 months**								
WHZ	-0.92 ± 1.05	-0.86 ± 0.96	-0.92 ± 1.00	-0.90 ± 1.00	-0.98 ± 1.04	-0.87 ± .95	-0.82 ± 0.99	-0.89 ± 0.99
HAZ	-1.89 ± 0.96	-1.92 ± 0.92	-1.99 ± 1.05	-1.94 ± 0.98	-1.96 ± 1.07	-1.99 ± 1.09	-2.03 ± 1.06	-1.99 ± 1.07
WAZ	-1.57 ± 1.00	-1.55 ± 0.94	-1.63 ± 1.06	-1.58 ± 1.00	-1.66 ± 1.09	-1.60 ± 1.04	-1.57 ± 1.03	-1.61 ± 1.05
**At 24 months**								
WHZ	-0.89 ± 0.96	-0.94 ± 0.93	-0.91 ± 0.96	-0.91 ± 0.95	-0.89 ± 1.00	-0.92 ± 0.91	-0.86 ± 0.95	-0.89 ± 0.95
HAZ	-1.91 ± 0.95	-1.92 ± 0.91	-1.99 ± 1.02	-1.94 ± .96	-1.99 ± 1.01	-2.02 ± 1.08	-2.06 ± 1.00	-2.03 ± 1.03
WAZ	-1.64 ± 0.96	-1.68 ± 0.89	-1.69 ± 1.02	-1.68 ± .96	-1.69 ± 1.05	-1.72 ± 1.02	-1.70 ± 0.98	-1.71 ± 1.02
**At 54 months**								
WHZ	-1.28 ± 0.84	-1.28 ± 0.83	-1.27 ± 0.81	-1.28 ± 0.82	-1.36 ± 0.87	-1.25 ± 0.78	-1.24 ± 0.81	-1.28 ± 0.82
HAZ	-1.44 ± 0.87	-1.49 ± 0.83	-1.55 ± 0.92	-1.49 ± 0.87	-1.52 ± 0.94	-1.61 ± 0.98	-1.62 ± 0.91	-1.58 ± 0.94
WAZ	-1.71 ± 0.83	-1.74 ± 0.81	-1.78 ± 0.82	-1.74 ± 0.82	-1.81 ± 0.91	-1.79 ± 0.89	-1.79 ± 0.85	-1.80 ± 0.88

Data are means ± SD

**Abbreviations: **WHZ = Weight for Height Z-score, HAZ = Height for Age Z-score, WAZ = Weight for age Z-score; Fe30F = 30 mg iron and 400 μg of folic acid; Fe60F = 60 mg iron and 400 μg of folic acid; MMS = multiple micronutrients, 15 micronutrients including 30 mg iron and 400 μg of folic acid;

A longitudinal analysis of linear growth was performed using repeated-measures analysis (Table 3). There was no interaction between food and micronutrient supplementation on linear growth. There was a tendency of better linear growth in early invitation food group (in comparison with usual invitation, p = 0.07) and impaired linear growth in MMS group (as compared to the Fe60Fgroup, p = 0.09). Early invitation to prenatal food supplementation to pregnant mothers resulted in significantly reduced proportion of stunting (average difference across 16 measurements 0-54 months 4.5 percent units, 95% CI = 1.2 to 7.8 percent units, p = 0.01). MMS supplementation resulted in significantly more stunting in comparison with Fe60F (average difference across 16 measurements 0-54 months 4.8 percent units, 95% CI = 0.8 to 8.9, p = 0.02).

**Table 3:** Effects of prenatal food and micronutrient supplementations on linear growth (16 anthropometric assessments) from birth to 54 months of age

Randomized intervention		n	HAZ mean (95% CI)	P-value^1^	Stunting, mean% (95% CI)	P-value^1^
Food supplementation	Early invitation (E)	839	-1.52 (-1.58, -1.46)^2^	0.07	30.8 (28.5-33.1)^4^	0.01
	Usual invitation (U)	795	-1.60 (-1.66, -1.54)		35.3 (32.9-37.7)	
Micronutrient supplementation	Fe30F	518	-1.51 (-1.59, -1.44)	0.09	31.8 (28.8-34.7)	0.04
	Fe60F	566	-1.55 (-1.62, -1.48)		31.3 (28.5-34.1)	
	MMS	550	-1.63 (-1.70, -1.55)^3^		36.1 (33.3-39.0)^5,6^	
Interaction Food*Micronutrients	E-Fe30F	258	-1.47(-1.58, -1.36)	0.99	29.9 (25.7-34.1)	0.94
	E-Fe60F	291	-1.51 (-1.61, -1.41)		28.7 (24.7-32.6)	
	E-MMS	290	-1.59 (-1.69, -1.49)		33.9 (29.9-37.8)	
	U-Fe30F	260	-1.55 (-1.66, -1.44)		33.6 (29.5-37.8)	
	U-Fe60F	275	-1.59 (-1.69, -1.48)		33.9 (29.9-38.0)	
	U-MMS	260	-1.67 (-1.78, -1.56)		38.4 (34.2-42.6)	

General lineal models, repeated-measures analyses, n = 1634.

**Abbreviations: **HAZ = Height for Age Z-score; Fe30F = 30 mg iron and 400 μg of folic acid; Fe60F = 60 mg iron and 400 μg of folic acid; MMS = multiple micronutrients, 15 micronutrients including 30 mg iron and 400 μg of folic acid;^1 ^Test of between-subjects effects;^2^Pairwise comparison with usual invitation, P = 0.07;^3^Pairwise comparison with Fe30F, P = 0.03;^4 ^Pairwise comparison with usual invitation; P = 0.01^5^Pairwise comparison with Fe30F, P = 0.04;^6^Pairwise comparison with Fe60F, P = 0.02

Weight-for-age and weight-for height Z-scores as continuous and dichotomous variables were also compared in repeated-measures analysis, and we found neither any interactions between food and micronutrient groups nor any significant main effects on growth (data not shown).

**Figure 2:**
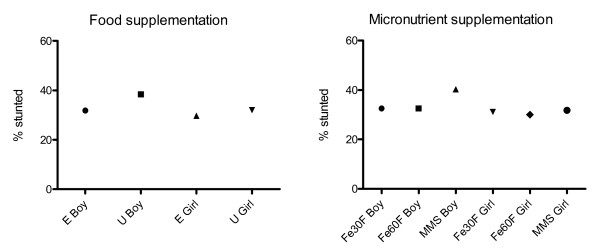
**Repeated measures analyses showing effects of prenatal food and micronutrient supplementations on linear growth (16 anthropometric assessments) by sex, from birth to 54 months of age**. Dots represent mean%. p-values for the contrasts from the repeated-measures analysis. E Boy vs. U Boy, p = 0.01; Fe60F Boy vs. MMS Boy, p = 0.01. E Girl vs. U Girl, p = 0.31; Fe60F Girl vs. MMS Girl, p = 0.53. E = Early invitation to food supplementation, U = Usual invitation to food supplementation, Fe30F = 30 mg iron and 400 μg of folic acid; Fe60F = 60 mg iron and 400 μg of folic acid; MMS = multiple micronutrients, 15 micronutrients including 30 mg iron and 400 μg of folic acid.

The effect of early vs. usual invitation to food supplementation on frequency of stunting was significantly shown for boys (average difference 6.5 percent units, 95% CI = 1.7 to 11.3 percent unit, p = 0.01), but not for girls (average difference 2.4 percent units, 95% CI=-2.2 to 7.0 percent unit, p = 0.31), Figure 2. The increased proportion of stunting in the MMS group was also more expressed among boys (average difference in comparison with Fe60F 7.8 percent units, 95% CI = 2.0 to 13.6, p = 0.01) as compared to girls (average difference 1.8 percent units, 95% CI=-3.8 to7.3, p = 0.53).

**Figure 3:**
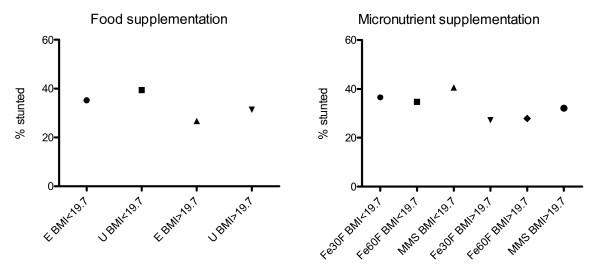
**Repeated measures analysis highlighting the effects of prenatal food and micronutrient supplementations on linear growth (16 anthropometric assessments) by maternal BMI (median) from birth to 54 months of age**. Dots represent mean%. p-values for the contrasts from the repeated-measures analysis E BMI < 19.7 vs. U BMI < 19.7, p = 0.09; Fe60 BMI < 19.7 vs. MMS BMI < 19.7, p = 0.05 E BMI > 19.7 vs. U BMI > 19.7, p = 0.05; Fe60 BMI > 19.7 vs. MMS BMI > 19.7, p = 0.13 E = Early invitation to food supplementation, U = Usual invitation to food supplementation, BMI = body mass index; Fe30F = 30 mg iron and 400 μg of folic acid; Fe60F = 60 mg iron and 400 μg of folic acid; MMS = multiple micronutrients, 15 micronutrients including 30 mg iron and 400 μg of folic acid.

The analysis was also stratified by maternal BMI using the median BMI 19.7 as cut-off level. Among mothers with higher BMI (BMI > = 19.7) stunting was less frequent (difference 4.6 percent units, 95% CI = 0.1 to 9.1 percent unit, p = 0.05) in early invitation food group (in comparison with usual invitation), while this was not significant among mothers in the lowest half of the BMI distribution (difference 4.3 percent units, 95% CI=-0.6 to 9.2 percent unit, p = 0.09). The increased occurrence of stunting in the MMS group (in comparison with Fe60F) was found only among mothers in the lowest half of the BMI distribution (difference 5.9 percent units, 95% CI=-0.1 to 11.8 percent unit, p = 0.05), Figure 3.

## Discussion

In this large randomized MINIMat trial in Bangladesh, children born to mothers receiving an early invitation to food supplementation were less likely to be stunted during the first five years of life. Children born to mothers who received multiple micronutrients rather than the standard iron-folate program, however, had a higher occurrence of stunting during the first five years of life: these effects were primarily in boys.

In MINIMat trial, the interviewers were unaware of the intervention groups and the mothers were unaware of their micronutrient supplement. By design, there was a difference in the number of food supplements of 18,000 kcal (30 packets) between the early and usual invitation groups. Allocation to food and micronutrient supplementation was according to MINIMat protocol, and masking was maintained for the micronutrient supplementation groups until the completion of intention-to-treat analysis. There was no difference among the groups in loss to follow-up, although there were minor differences in the number of capsules consumed across the groups. The pregnant mothers received almost 4 months of daily micronutrient supplements.

Sex differences in fetal growth are reported [22], but the underlying mechanisms are poorly understood and may reflect both genetic (cell division among male embryos occurs more rapidly than in female embryos [23]) and environmental factors (boys are often given preference in food allocation within the family in Bangladesh [24]). These observations suggest males may be more responsive to growth-promoting influences. The differences in stunting persisted until the last follow-up assessment at 54 months. Nevertheless, there was a trend of better linear growth in the infants in the early food group throughout the follow-up until the fifth year of life. The results indicate that early invitation of prenatal food supplementation had a positive effect on post- natal infant/child growth.

Early food supplementation had an effect on early child growth. In Indonesia, maternal nutrition during pregnancy influenced the growth of offspring in early childhood, and high-energy supplementation produced less stunting throughout the first five years [12]. In this study, there were more stunted children in the MMS group than in the iron+folic acid supplementation group. The reason prenatal MMS increased the risk of stunting is unclear, as a non-randomized, non-blinded trial in Vietnam found MMS during pregnancy to be an important intervention for reducing stunting in children [14]. In this Bangladeshi sample, MMS (rather than 30 mg or 60 mg iron) did not appear to benefit child growth, and this could have perinatal causes, such as potential interactions among nutrients producing adverse outcomes [25]. In Indonesian infants [26], single supplementation with either iron or zinc improved growth, yet combined supplementation with iron and zinc had no effect on child growth or development. Other possible reasons for prenatal MMS to increase the risk of stunting could include low doses of micronutrients (i.e, recommended daily dietary allowance) being insufficient for influencing fetal development [27]; the effects of the endocrine system on uterine sensitivity on long-term child growth [28,29]; and post natal causes, such as feeding practices, specifically breast feeding and complementary feeding, and morbidity. In a meta-analysis [30] comparing MMS with a placebo or no supplementation groups, there were reductions in anemia in pregnant women, low birth weight, and small-for-gestational age, but there was no greater response when MMS was compared to iron+folic acid.

The causes of stunting are complex and involve poor quality diet, breastfeeding practices, long-term burden of infectious disease morbidity, and chronic adverse environmental exposure; these factors are difficult to measure. The biological basis for the effects of multiple micronutrient supplements and the role of specific micronutrients in human biology is a complex mechanism [31,32]. Multiple micronutrients are needed for gene regulation, embryogenesis, and fetal development, and influence birth and infancy [31,33]. Thus, adequate availability of many micronutrients and improvement in body storage could affect the physiological and anatomical development of the fetus and be associated with the homoeostasis needed in infants to withstand adversity during birth and early infancy. A deeper understanding of the mechanisms is clearly needed. Thus, amelioration of multiple micronutrient deficiencies in women with insufficient macronutrient intake could enhance physiological, endocrine, or metabolic processes in infants, or optimize the anatomical structures that promote survival without changing total body mass; sufficient macronutrient intake along with improved MMS status could improve body mass, and therefore, survival.

The World Health Organization (WHO) recommends iron and folic acid supplementation to women during pregnancy as part of routine antenatal care. The effect of multiple-micronutrient supplementation with iron and folic acid supplementation was assessed, and we observed no significant added benefit of multiple-micronutrient supplements over iron and folic acid supplementation for post-natal growth. There is a lack published data presenting similar findings of more stunted children in a maternal MMS group than in iron+folic acid supplementation groups. This study was conducted in a low-income country, and further studies in different populations are needed to assess the consistency of the findings. The study was not designed to examine the potential risks of excess micronutrient supplementation or to examine potential adverse interactions between the micronutrients, which need addressing in future studies. As an early start to supplementation during pregnancy is beneficial for early child growth, future food supplementation during pregnancy should be implemented at the beginning of pregnancy. The randomized, double-blind design was a major strength of this study. As a placebo group could not be added due to ethical reasons, it is not appropriate to conclude micronutrient supplementation does not have any beneficial effect on birth outcome or early child growth.

It may be pertinent to ask the effect size of different nutrition interventions that improve child growth, particularly stunting. Our data suggest that early food supplementation during pregnancy had a positive impact on preventing stunting. Effects of nutrition-related interventions on stunting in 36 country analysis showed that nutrition education and counseling of complementary feeding and other supportive strategies could reduce stunting by 19.8% at 12 months, 17.2% at 24 months, and 15.0% at 36 months [34]. In our study, we did not have any interventions on complementary feeding education or counseling. Earlier study done in Matlab in the specific population has shown that the overall rate of stunting was 63% in 2002 [35]. In our study stunting was reduced by 13% (difference: 35.3-30.8 = 4.5 unit, reduce stunting by 12.7%) when food supplementation could start during early stage of pregnancy compare to late start and could be of major public health importance. This study provided evidence of the potential benefit of the government-supported nutrition program even it targeting to women with BMI < 18.5 may be questioned. Community facilitators educate the pregnant women and encourage them to visit the community nutrition centers to obtain the supplements. Due to the novel design of the MINIMat study, and the use of the existing health system supported by non-governmental organizations, the results are relevant for public-health planners and policymakers. In addition, more attention should be given to the issues of optimal delivery vehicles (including supplements, fortified foods), program efficacy and effectiveness, and the target (i.e., whether stunted children should be targeted). For sustainable solutions, efforts should continue to adopt and improve locally available and affordable food-based approaches.

## Conclusions

Early food supplementation in pregnancy reduced the occurrence of stunting during 0-54 months in boys, but not in girls, and prenatal MMS increased the proportion of stunting in boys. The results of this study indicate food supplementation to women during early pregnancy reduces early childhood malnutrition, especially stunting. These effects on postnatal growth suggest programming effects in early fetal life.

## Competing interests

The authors declare that they have no competing interests.

## Authors' contributions

LÅP, IK and AIK: study design; LÅP, SEA, and EAF: designed the MINIMat study; AIK, KÅM, DSA, MU, SEA, and LÅP: supervised data collection; AIK, LÅP, and EAF: statistical analysis; LÅP: supervised data analysis and interpretation; AIK: writing of first draft of the manuscript. All authors contributed to subsequent discussions and revisions. All authors read and approved the final manuscript.
